# Developmental Change in the Function of Movement Systems: Transition of the Pectoral Fins between Respiratory and Locomotor Roles in Zebrafish

**DOI:** 10.1093/icb/icu014

**Published:** 2014-04-17

**Authors:** Melina E. Hale

**Affiliations:** University of Chicago, Organismal Biology and Anatomy

## Abstract

An animal may experience strikingly different functional demands on its body’s systems through development. One way of meeting those demands is with temporary, stage-specific adaptations. This strategy requires the animal to develop appropriate morphological states or physiological pathways that address transient functional demands as well as processes that transition morphology, physiology, and function to that of the mature form. Recent research on ray-finned (actinopterygian) fishes is a developmental transition in function of the pectoral fin, thereby providing an opportunity to examine how an organism copes with changes in the roles of its morphology between stages of its life history. As larvae, zebrafish alternate their pectoral fins in coordination with the body axis during slow swimming. The movements of their fins do not appear to contribute to the production of thrust or to stability but instead exchange fluid near the body for cutaneous respiration. The morphology of the larval fin includes a simple stage-specific endoskeletal disc overlaid by fan-shaped adductor and abductor muscles. In contrast, the musculoskeletal system of the mature fin consists of a suite of muscles and bones. Fins are extended laterally during slow swimming of the adult, without the distinct, high-amplitude left-right fin alternation of the larval fin. The morphological and functional transition of the pectoral fin occurs through juvenile development. Early in this period, at about 3 weeks post-fertilization, the gills take over respiratory function, presumably freeing the fins for other roles. Kinematic data suggest that the loss of respiratory function does not lead to a rapid switch in patterns of fin movement but rather that both morphology and movement transition gradually through the juvenile stage of development. Studies relating structure to function often focus on stable systems that are arguably well adapted for the roles they play. Examining how animals navigate transitional periods, when the link of structure to function may be less taut, provides insight both into how animals contend with such change and into the developmental pressures that shape mature form and function.

## Introduction

An animal must be able to function effectively throughout its development. Often a given function is maintained as the systems that underlie it grow and transform (e.g., Henning 1981; Blumberg-Feldman and Eilam 1995; Richard and Wainwright 1995; Hale 1999; Hernandez 2000). However, in some cases, a morphological or physiological system must serve discretely different functions at particular points in life history. This later developmental trajectory is less explored, yet likely common. A morphological example of such functional transition is the adhesive pad on the snout of larval longnose gar (*Lepisosteus osseus*) that allows the young animal to stick to the substrate (Goff 1984) for protection, but disappears later in life as the animal develops greater ability to forage and evade predators. Neural control can also mediate such functional changes; for example, components of motor activity are discretely different between the suckling motor pattern of infant rats (*Rattus norvegicus*) and the feeding motor patterns in adults (Westneat and Hall 1992), despite commonality in the underlying morphological system. In the evolution of such distinct functional systems of sub-adults, a balance must be struck between expression of morphology and physiology that will allow appropriate behaviors in immature stages and the need to generate the body plan and behaviors of the adult form.

It is unclear how stage-specific, and possibly conflicting, pressures on function impact morphology, physiology, and behavior. Developmental change can be mediated through a range of mechanisms. Morphologically and physiologically, new cells, tissues, or structures can be added, or existing systems can be retooled. Body size often also changes significantly, impacting internal physiology and biomechanics. Size affects the physics of an organism’s movement in surrounding media as well as its broader behavioral strategies, such as how it seeks refuge and uses food resources. The scaling of body elements also varies and allometric growth, particularly in immature organisms, can focus available resources on morphological regions or physiological processes of particular functional relevance at a given stage of life history. Behaviorally, alternative patterns of motor control can be employed in order to generate different behaviors with the same morphology. On the foundational development of morphology, physiology, and motor control, ecological factors add additional dimensions for change (e.g., Rombough 1988; Barrionuevo and Burggren 1999) and provide feedback that may modulate developmental processes and influence behaviors.

Here I will use the pectoral fins of ray-finned fishes, specifically of the zebrafish species (*Danio rerio*), as a case study to explore how a system functions through life history and to analyze the relationship of function to adaptations of underlying systems. In recent years, work on pectoral fins has explored their morphology, physiology, and function at a range of life-history stages and from multiple perspectives. I review these data to argue for a larval stage-specific functional morphology of fins. The life-history changes of the pectoral fin provides a framework for examining broad questions of how periods of transition between stable states are navigated through an animal’s development.

## Morphology and function of pectoral fins in adult fishes

As we explore the development of the pectoral fin system of fishes, the endpoint of the adult form and its function provides an important context for considering change. The pectoral fins of adult fishes have been broadly studied within the context of swimming (e.g. Lindsey 1978; Gibb et al. 1994; Westneat 1996; Drucker and Jensen 1997) and features both of morphology and of movement have been associated with swimming performance. In many species, pectoral fins are the primary propulsors through a wide range of swimming speeds and they have also been shown to be critical in braking and maneuvering in other taxa (e.g., Lauder et al. 2006; Higham 2007). The movement of the pectoral fins results in production of force through interaction with the fluid environment. Kinematic studies have examined the relationship between the movement of fins and swimming performance, demonstrating that fishes control the pattern of fin movement (e.g., Gibb et al. 1994; Walker and Westneat 2002) and fin-beat frequency (e.g., Gibb et al. 1994; Walker and Westneat 2002) to vary swimming speed. Several juvenile fishes additionally have been shown to use two fin-based, locomotor gaits, alternating and synchronous coordination patterns, at, respectively, slower and faster speeds (Hale et al. 2006).

The structure of the mature pectoral fin has been shown to be adapted to the fins roles of the fins in swimming. The shape of the pectoral fin greatly impacts interactions with the fluid around it and considerable variation in shape is evident among species and is associated with locomotor mode and performance (e.g., Blake 1981; Walker and Westneat 2002). Walker and Westneat (2002) showed that fish with fins of high aspect ratio (wing-shaped) have improved cruising performance. They suggest that those with fins of lower aspect ratio (more rounded) have advantages in high-acceleration starts and maneuvers, and improved stability during hovering and slow swimming. In addition, the stiffness of the fins also can vary and has been shown to impact performance in tests with engineered models (Tangorra et al. 2010). The stiffness of fin rays and the shape of fins can be actively controlled to modulate movement and interactions with fluids (e.g., McCutchen 1970; Geerlink and Videler 1987; Lauder et al. 2006; Alben et al. 2007). Comparative examination of the skeletal and muscle morphology of the fin also shows considerable variation among species and reveals associations with mode and performance of swimming (Thorsen and Westneat 2005).

While swimming-related movements may be the predominant functions of pectoral fins across fishes, a wide range of specializations for alternative functions have evolved as well. For example, the morphology and mechanics of the pectoral fins of longhorn sculpins are adapted for benthic station-holding (Taft and Taft 2012). The pectoral fins of sea robins include three free fin rays that have been modified as sensory substrate probes used in foraging (Bardach and Case 1965). In other species, locking spines of the pectoral fins have developed as defense (e.g., Hoogland et al. 1956). The broad range of morphologies and functions exhibited by the pectoral fins shows these fins to be an adaptable and diverse system. Major questions remain as to how such diversity arises through development and evolution. Below I focus on a specific aspect of this: how a pectoral fin is organized and functions early in life history and changes as it develops toward the adult condition.

## Morphology of the pectoral fins of larval and juvenile fishes

The pectoral fins of larval fishes differ markedly from those of adults both in gross structural features and in their patterns of movement. While there is little comparative work on the ultrastructure of the musculoskeletal components of the fin, images from several phylogenetically distant taxa (herring [*Clupea harengus*] [Ehrlich et al. 1976]; torafugu [*Takifugu rubripes*] [Asaduzzaman et al. 2013]; and zebrafish [*D. rerio*] [e.g., Grandel and Schulte-Merker 1998; Thorsen and Hale 2005]) suggest that the anatomy shares basic organization among diverse groups. The pectoral fins of the larval zebrafish, with the molecular and genetic accessibility afforded to the zebrafish as a model species, have been studied from a broad range of perspectives and provide a key example of the morphology and function of the larval fin as well as its development, and I will focus on that species. Here I review morphological evidence that supports the idea that the pectoral fin demonstrates adaptations specific to the larval stage.

In the larval zebrafish hatching most commonly occurs between 48 and 72 h post-fertilization, when fish are about 3.1–3.5 mm in length (Kimmel et al. 1995), although the timing of hatching and body length at hatching vary with environmental temperature and other factors. The yolk sac can sustain larval zebrafish through several days of early post-hatching development; however, exogenous feeding begins fairly soon after hatching and is critical to the health of the fish by the end of its first week. During embryonic development, the pectoral fin bud forms at about 24 h post-fertilization; by 48 h the fin has elongated and its skeleton has begun to chondrify (Grandel and Schulte-Merker 1998; Yelon et al. 2000) so that by the time of hatching the larval fin has taken shape.

**Fig. 1:**
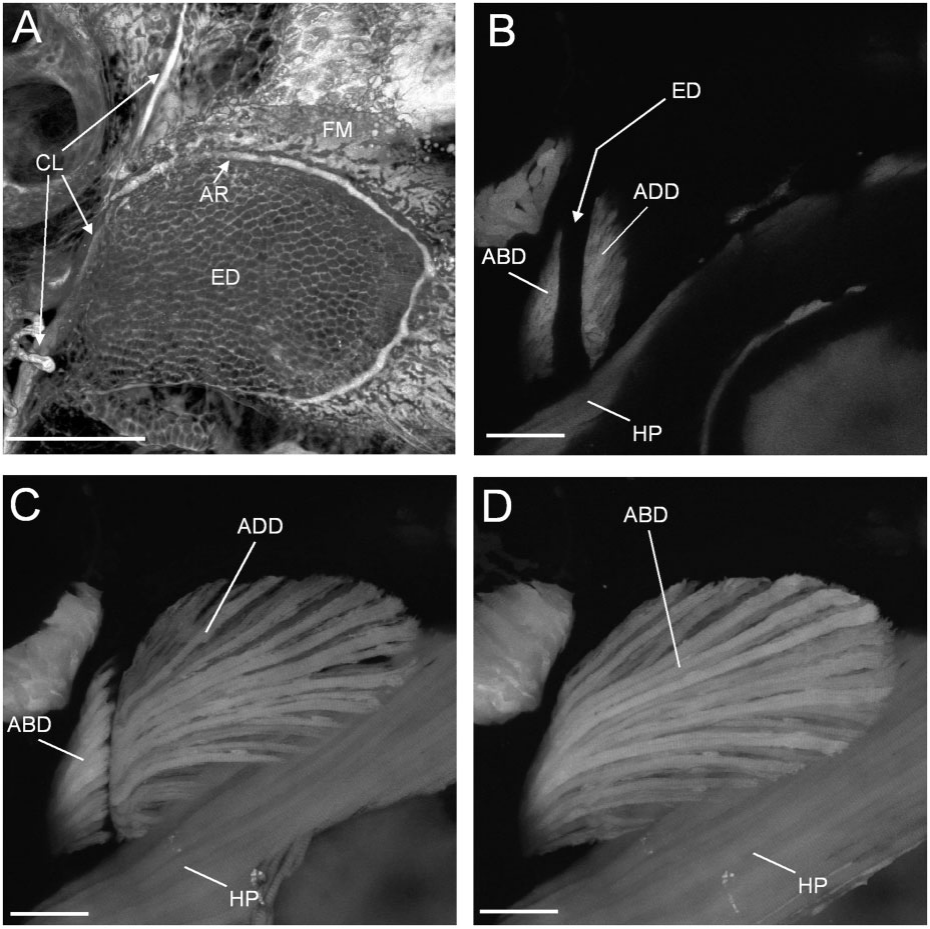
Confocal images of the endoskeletal disc and pectoral fin musculature in 5 dpf larval zebrafish. Lateral view confocal slices (B–D) from the same 3D stack. (**A**) The endoskeletal disc (ED), distal fin membrane (FM), and basal cleithrum (stained *in vivo* with calcein green). AR is the pectoral fin artery that delimits the endoskeletal disc. (**B**) Abductor (ABD) and adductor (ADD) musculature in cross-section near their proximal end. The endoskeletal disc that separates would be in the space indicated. Hypaxial musculature (HP) lies medially to the fin. (**C**) Adductor muscle and a section through the abductor muscles. (**D**) Extent of the abductor in lateralmost view. Scale bars = 100 μm. Reprinted with permission from Thorsen and Hale (2005).

Post-hatching, two phases of skeletal development have been distinguished in the pectoral fin (Grandel and Schulte-Merker 1998). The first stage includes a distinctive skeleton that is specific to the larva. By 4 days post-fertilization, an endochondral disc has formed proximal to the distal membrane and continuous with the developing scapulocorocoid at the base of the fin (Grandel and Schulte-Merker 1998) (Fig. 1A). The endoskeletal disc is a cartilaginous, plate-like structure that persists through the second week of development. During this time, cells of the disc divide once to create a bilayered structure; subsequent expansion is through growth of the existing cells, rather than by cell division. In the second phase of development, the endoskeletal disc is remodeled; with regions of the plate degrading to leave the precursors of the cartilage of the proximal radials, the skeletal elements that will articulate with the fin rays distally. The radials, fin rays, and bones at the base of the fin develop as endochondral or dermal bone during this period (Cubbage and Mabee 1996; Grandel and Schulte-Merker 1998). In addition, a large-scale rotation of the fin occurs so that it transitions from being oriented vertically against the body in the larva to taking a more horizontal position in the adult (Grandel and Schulte-Merker 1998; Thorsen and Hale 2005).

The development of the endoskeleton suggests a larva-specific morphological phase, distinct from the immature morphology of the forming pectoral fin of the adult. Grandel and Schulte-Merker (1998) noted that the initial phase of pectoral fin development contrasts strikingly to that of the pelvic fins. While the endoskeletal elements of the pelvic fin initially form as models for ossification, the endoskeleton of the pectoral fin is discretely different from later morphology, developing and persisting as one continuous disc during the first 2 weeks of development. This process of development appears common for pectoral fins of actinopterygian fishes (reviewed by Grandel and Schulte-Merker 1998). It was suggested that this morphology supports use of the pectoral fins in locomotion by larvae in an early, “premature” developmental state.

The musculature of the larval fin is also distinct from that of the adult. In the fin of the 5 dpf larval zebrafish, the musculature is composed of two fan-shaped sheets of muscle, the abductor and adductor (Fig. 1B–D), each only one or two muscle fibers thick (Thorsen and Hale 2005). The fibers are closely associated at the base of the fin and splay out toward the distal margins. Throughout larval development, this morphology is retained while the numbers of muscle fibers increase. From ∼5 mm total body length (corresponding to the second phase of pectoral fin development [Grandel and Schulte-Merker 1998]) significant change occurred in these muscle. The abductor and adductor grow, increasing in numbers of fibers, each subdivides through their thickness and along their span to form six distinct muscles from each of the two precursors; the angles of the fibers change and the muscles connect to the growing skeleton and associated connective tissues, both at the base of the fin and at the proximal ends of the developing rays (Thorsen and Hale 2005).

**Fig. 2:**
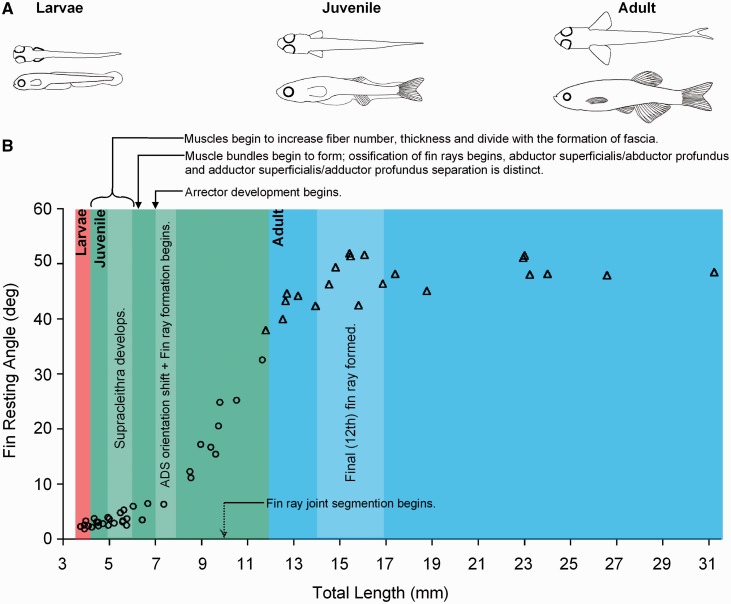
Development of pectoral fin position in zebrafish. (**A**) Through development the pectoral fins transition to a laterally splayed resting position. (**B**) Change in resting angle of the pectoral fin, plotted against total length of the body and overlayed with changes in the musculoskeletal system. Additionally, general observations on movement of the fins are shown. Circles indicate coordination of the fins with the axis during slow swimming; triangles represent the pattern of slow swimming in adults, when pectoral fins splayed laterally. Reprinted with permission from Thorsen and Hale (2005).

Mapping muscle morphology onto the change in angle of the fin as it rotates from vertical to horizontal orientation (Fig. 2), we found that prior to the shift in fin angle (occurring from ∼4–6 mm TL), abductor and adductor muscles increase in fiber number and overall muscle thickness. By 7 mm discrete superficial and deep fin muscles can be identified, muscle bundles can be distinguished along the span of the fin and the arrector muscles develop. Types of muscle fibers also are changing, with fibers staining as a fast-fiber type up to ∼7 mm and developing a slow-fiber component after that (Patterson et al. 2008). Toward the end of this period, the angle of the fin shifts rapidly. By ∼12 mm TL, developmental phase 2 ends with the establishment of the mature morphology.

## Movement and function of the larval zebrafish’s pectoral fin

**Fig. 3:**
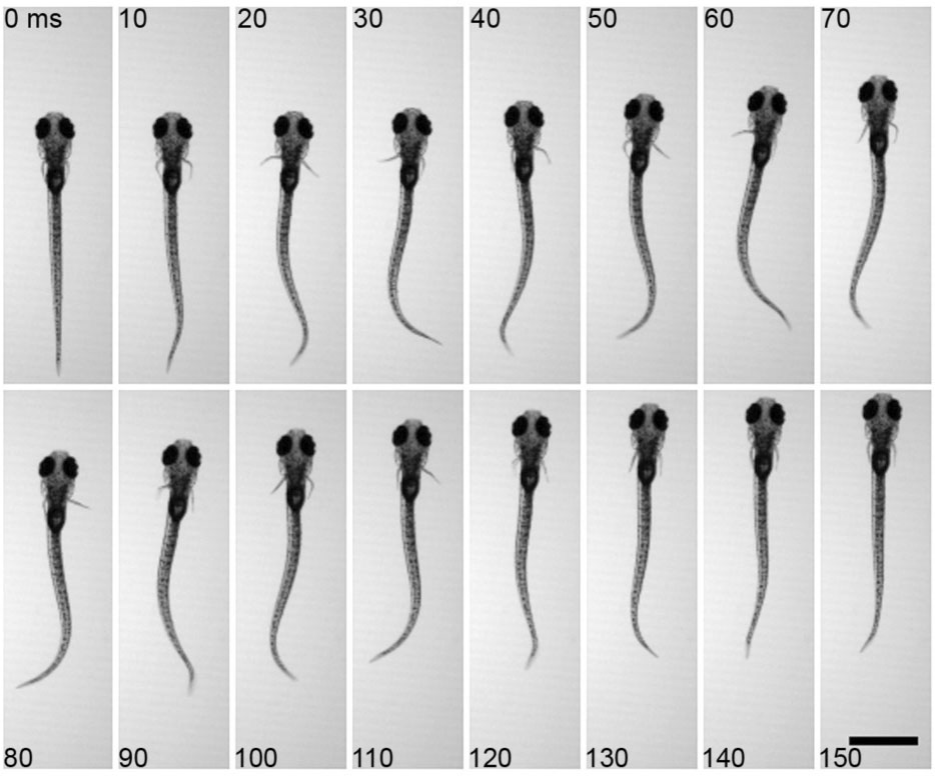
Typical movements of the pectoral fins and the body axis during slow swimming of larval zebrafish. Fins alternate between the left and right sides and in coordination with axial bending. Note bending of the fin during abduction (e.g., on right side at 40 ms), Scale bar, 1 mm. Reprinted with permission from Green et al. (2011).

As with morphology, larval zebrafish demonstrate stage-specific fin movement and function. Larval zebrafish actuate their pectoral fins in coordination with the body axis during slow swimming. At the initiation of a bout of swimming, the fins commonly abduct together but quickly fall into an alternating pattern between left and right sides (Fig. 3). Pectoral fins are also able to beat, in similar alternating bouts, without the participation of the body axis, although this is uncommon under normal conditions (Green et al. 2011). Pectoral fin movements by larvae, unlike those of adults, occur over a relatively narrow range of fin beat frequencies at ∼30 Hz. During fast, axial swimming, the fins do not alternate but are held next to the body (Thorsen et al. 2004; Müller and van Leeuwen 2004; Green et al. 2011) with active adduction (Green and Hale 2012).

**Fig. 4:**
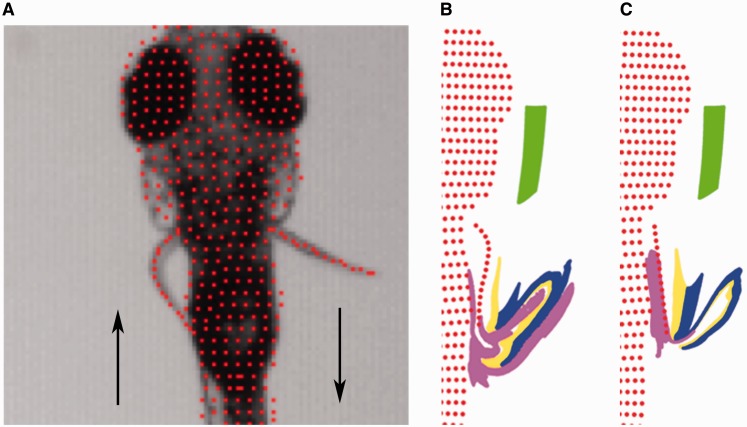
Fin bending and modeling of fluid movement associated with the pectoral fins. (**A**) Fin movement during abduction and adduction of the pectoral fins demonstrates asymmetry in bending between these phases of movement. (**B**) Flow modeled in a representation of fin morphology and movement of larval zebrafish. (**C**) Flow in a manipulated model in which the pectoral fin remains straight as they are abducted and adducted though the fin beat cycle. Fin bending during abductions increases fluid-folding in larval zebrafish, suggesting that it is adapted to support respiratory exchange (Green et al. 2013). Images are an output from modeling performed by M. H. Green and O. Curet in association with Green et al. (2013).

Focusing on the movement pattern of an individual fin we see that, although the structure of the larval pectoral fin appears rather symmetrical, both superficially and in its musculoskeletal structure, its movement is strikingly asymmetric. During the abduction phase of the fin-beat cycle, the pectoral fin bends midway along its proximodistal axis so that the distal end curves backward (Fig. 4A). The abducting fin curves back at a consistent position ∼44% along the length of its proximal to distal axis at a consistent phase (Green et al. 2013). During the adduction phase of the fin-beat cycle, the fin remains nearly straight with no comparable local bending. Local bending could be produced by differences in motoneuron activity in the two phases of movement but neurophysiological recordings suggest that the difference in curvature of the fin is not actively controlled. No obvious difference in the activity pattern of individual motoneurons or in multiunit ventral root recordings suggest an active mechanism, either for generating fin bending during abduction or for stiffening during adduction and thus we hypothesize that the asymmetry is generated passively through the mechanics of tissues (Green and Hale 2012).

**Fig. 5:**
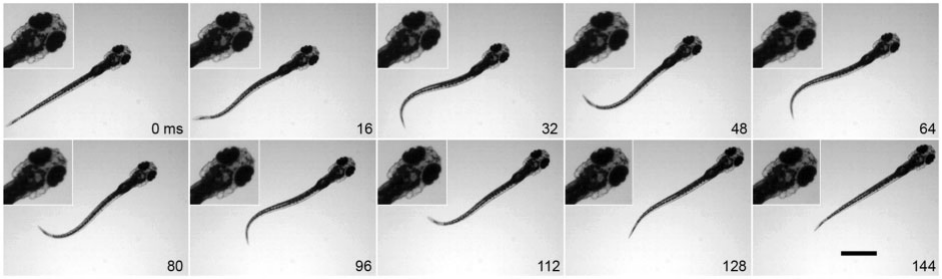
Slow swimming by a finless larval zebrafish. A full bout of swimming at axial frequencies typical of slow swimming of larval zebrafish with fins. The magnified images of the head illustrate the stability of the finless fish in roll and yaw. Scale bar = 1 mm. Reproduced with permission from Green et al. (2011).

Potential roles of the pectoral fins in locomotion have been investigated, using experimental approaches (Green et al. 2011) and computational fluid dynamics (Green et al. 2013), and focusing on the behavioral context of slow, forward swimming. Morpholino injections that blocked translation of fgf24, a gene that participates in initiation of forelimb development (e.g., Fischer et al. 2003) prevented formation of the pectoral fins while leaving other aspects of morphology comparable to the typical larva (Green et al. 2011). The pattern of axial movement and the performance of slow swimming were not significantly different between morpholino-injected finless fish and normal fish (Fig. 5). There was no difference in the stability of the body in either roll or yaw and the fish did not appear to compensate for loss of the fins with changes in axial movements. Thus, although coordinated with the axis, the pectoral fins of larvae do not appear to function in generating thrust or stabilizing the body during slow swimming.

An alternative hypothesis for the function of pectoral fins in larvae is that the fin is a respiratory structure (Hunter 1972; Weihs 1980; Osse and van der Boogart 1999). For small organisms, like the larval zebrafish, the fluid boundary layer is thick compared with body size. This impacts fluid movement near the surface of the body. In larval zebrafish, the skin is the primary location for exchange of ions and gas, including oxygen exchange for respiration, and it was proposed that larvae may use the pectoral fins to exchange oxygen-depleted water near the body with distant oxygen-rich fluid. Dye-based flow visualization and computational fluid dynamics demonstrated that, indeed, the fins do pull fluid distant from the body toward the trunk and move fluid in the boundary layer away from the side of the body (Green et al. 2011, 2103). In this way they fold fluid along the body (Fig. 4B), a mechanism for chaotic mixing under viscous conditions (Ottino 1989; Strook et al. 2002; Ottino and Wiggins 2004).

Although features of larval pectoral fin morphology suggest the presence of a distinctive organization in the larval stage, it is difficult to address whether fin movement in larvae is adapted to function in gas and/or ion exchange. One feature of kinematics that suggests the fins are specialized for movement and mixing of fluid is the fin bending in abduction, observed experimentally and discussed above. To address whether that bending is important for exchange at the skin, computational modeling was used to compare effectiveness of a normally bending and a straight fin in moving fluid along the side of the body (Fig. 4C). Bending of the fin during abduction improved movement of fluid near the body when compared with the straight fin, suggesting that its movement is adapted for the function of the fin in respiration.

Examining behavior of the pectoral fins in response to different, or differently perceived, oxygen levels provides ways to test the hypothesis of respiratory function. Green et al. (2011) explored whether this movement of the fin was associated with respiratory function by examining behavior of the fins and axis under different dissolved oxygen conditions. Decreasing the level of dissolved oxygen in the water resulted in a significant increase in the number of bouts of fin movement expressed during a given period of time. Changes were specifically observed in fin-only behavior, as opposed to axial swimming or coordinated fin and axial movement. van Rooijen et al. (2009) found a similar result but with a different approach. They identified lines of zebrafish with mutations in the *vhl* gene, which is part of the hypoxia response pathway. These animals perceive typical environmental conditions as hypoxic. The larvae of *vhl* mutants gulp water and beat their fins continuously, consistent with a behavioral response to hypoxia response behavior under the proposal that movement of the fins functions to generate respiratory flow.

Together these complementary data on morphology, movement, physiology, and mechanics argue that the fins are adapted to serve as respiratory structures during larval development. They support the idea that the pectoral fin goes through a distinct stage of musculoskeletal morphology in the first few weeks post-fertilization, during which time the fins generate bending and overall movement that is appropriate for respiratory function. Following comparative data of Grandel and Schulte-Merker (1998) that show commonalities in morphology among taxa, I suggest that this respiratory function of the pectoral fins will be common to many small fish larvae.

## Development of respiratory systems and transitions in morphology of the pectoral fin

If the fins of larvae have a significant role in respiration and are adapted to that function but the fins of the adult fish serve other functions and have specific adaptations to different roles, this implies a transition between these two stable life-history states in which the animal may not be well suited to either function. How is this transition accomplished? The adult zebrafish fin behaviors and functions have received less attention than those of the larvae. The mature pectoral fins move little during slow axial swimming and for the most part remain splayed laterally during slow forward swimming. Although they are actuated during turning and asymmetries in their movement are associated with direction of turn (personal observation, ME Hale), Danos and Lauder’s (2007) developmental analysis of maneuvering found no clear evidence that the pectoral fins played a significant role in maneuvering behaviors in the adult. However, they also suggest a more detailed analysis of the system is warranted. While roles of the mature pectoral fins of zebrafish remain ambiguous, it seems clear that they are not used for respiratory fluid movement or production of thrust during slow swimming.

The loss of their respiratory function is a key event in the development of the pectoral fins. Cutaneous exchange of gas and ions has been studied in depth in zebrafish. The role of the skin in exchange declines precipitously in the 3 weeks after fertilization. The relocation of exchange function from skin to gills has been examined in zebrafish with histology (Jonz and Nurse 2005, 2006) and by manipulating ventilation function (Rombough 2002). The gills appear to become necessary for ion exchange before they are required for gas exchange. The skin is sufficient for uptake of ions until about 7 days post-fertilization and is sufficient for uptake of oxygen until 14 dpf (reviewed by Rombough 2007). This slight offset between these systems is consistent with morphological development (Rombough 2002; Jonz and Nurse 2005, 2006) in which mitochondria-rich cells, associated with ion exchange develop in the gills prior to the development of secondary lamellae that are important for oxygen uptake. This and other data have led to the hypothesis that exchange of ions, not gas, is the most important functional pressure on gill development (e.g., Li et al. 1995; Rombough 1999; reviewed by Rombough 2007).

From the perspective of cutaneous respiration and the function of the pectoral fins, these data suggest that an association of the pectoral fins with moving fluid near the skin is more important for oxygen exchange than for ion exchange after ∼1 week of development. The pectoral fins appear to maintain their importance in respiration through the second week of development but decrease significantly in importance by the end of the third week. Such associations are both driven and complicated by concurrent changes in other body structures and in overall size. Increased size is likely the most important factor requiring gill-based respiration. In smaller animals with a higher surface area to volume ratio, the surface of the skin is able to meet the needs for exchange both of ions and of gas without gill function. In larger animals, ∼100 mg (Rombough and Moroz 1997), cutaneous exchange becomes limiting for oxygen uptake.

The timing of transition from cutaneous to gill-based respiration is associated with phase 2 of fin development, when the fin increases in both its number and its specialization of muscle and skeletal elements (Grandel and Schulte-Merker 1998; Thorsen and Hale 2005). In addition, during this period the angle of the fin with respect to the body changes rapidly and away from its vertical orientation in the larva (Grandel and Schulte-Merker 1998; Thorsen and Hale 2005). While we have not examined what this change in angle means for movement of fluid, I suggest that this more mature lateral position of the fin may not be as effective at moving fluid along the body as the vertically oriented position observed in the larva. The relationship between structural and functional change in the gills and fins suggests that the morphological transitions in the pectoral fins of juvenile zebrafish are developmentally coordinated with the loss of respiratory function. These many changes both in role and in morphology raise the major question of how such a transitional structure is integrated into behavior.

## Transition in pectoral fin movement from the larval to the adult stage

During the juvenile period, change is not only in morphology and role of the pectoral fin, discussed above, but also in the motor activity that generates movement. Motor activity is underlain by the organization of neural circuits that drive movement, which may themselves be changing in morphology and functional ability. However, if we assume that the larval and young juvenile zebrafish have the ability to modulate basic patterns of limb movement throughout this period then we can imagine two levels of change: one is the change in musculoskeletal morphology of the fin occurring in the time-frame of months and the other is modulation of motor control that could occur within the time-frame of a behavior. This raises the question of whether, once the fins are no longer used significantly in respiration, changes in motor control alter movement of the early juvenile fin toward the adult pattern of actuation. Alternatively, fin morphology and motor control may develop together in lockstep or change in motor control could lag that of morphology. It is also possible, perhaps most likely, that different aspects of fin behavior change at different rates or at different points in morphological development.

To begin to explore movements of the pectoral fins of juvenile zebrafish, I examined slow swimming in zebrafish soon after the transition from fin-based to gill-based respiration is complete. I assessed basic features of slow swimming in four fish (10 trials per fish) at 33 days post-fertilization. The fish had an average body length of 7.1 ± 0.9 mm (mean ± SD). I used ImageJ (NIH; http://imagej.nih.gov/ij/) to examine videos recorded at 125 Hz with a Basler A504K high-speed video system (Basler Vision Technologies, Ahrensburg, Germany) and XCAP software (Epix, Buffalo Grove, IL, USA). As is typical for larval zebrafish, the animals performed slow swim bouts regularly in the filming dish (60 mm diameter Falcon petri dish; Fisher Scientific, Pittsburgh, PA, USA) and water from the fish’s home tank.

**Fig. 6:**
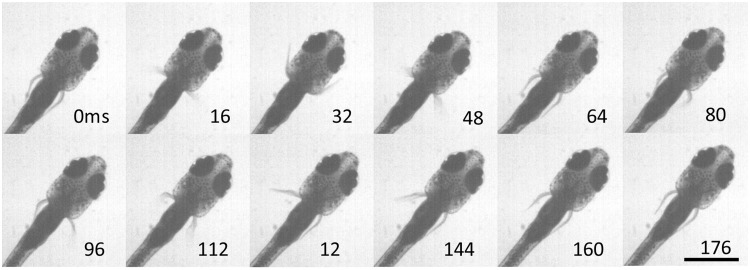
Typical movement of the pectoral fins during slow axial swimming in a juvenile zebrafish at 35 dpf. The series shows a full, synchronized fin beat cycle at initiation followed by a single fin-stroke on each side of the body. Scale bar = 1 mm.

Under our laboratory conditions, swim bouts in larval zebrafish (total length of 3.95 ± 0.16 mm, mean ± SD) included coordinated fin and axial movement lasting 147 ± 26 ms and with fin beat frequencies of 28.2 ± 3.5 Hz (Green et al. 2011) about approximately five fin beats per bout (Fig. 2). In the juvenile, swimming also involves the pectoral fins and body axis but the swim bouts include fewer fin beats (Fig. 6). All 40 trials included an initiating beat with both fins; in 11 bouts, this was followed by one fin beat on one side, in 23 bouts by one beat per side, and 6 bouts had a full beat per side plus one additional fin beat on the left or right, averaging 1.93 ± 0.32 cycles, with a full cycle of swimming considered to be a left fin beat and a right fin beat. Bouts lasted 202 ± 42 ms and fin beat frequencies averaged 12.7 ± 4.3 Hz. These data show that at this stage of development, after the gills take on primary respiratory responsibility, the fins maintain their alternating pattern of activity although the repeated cycles of movement are reduced.

These data indicate that an alternate motor program to that of the larva is not employed to switch the movement pattern of the pectoral fins to that of the adult once the role of the fins in respiration ends. They also demonstrate that the larval motor pattern is not retained until the mature morphology is established. Instead, an intermediate behavior is present, suggesting that the transitions in musculoskeletal morphology and in motor control are occurring together. It is possible that the observed movement pattern of the fin serves particular functions in the juvenile and this will need to be investigated. Danos and Lauder (2007) suggested that the pectoral fins of the later juvenile generate power for turning behavior but do not show a kinematic asymmetry associated with direction of movement. It would be interesting to explore how possible function in power generation develops between larvae and adults and whether it represents yet another functional stage for the pectoral fins.

## Modulating the developmental transition in respiratory function

In addition to genetically determined stability and change in function through life history, development is regulated in response to environmental factors. In the case of the transition of the pectoral fins from a larval respiratory function, one might expect feedback on levels of oxygen in the environment to modulate the timing or process of development. The oxygen environment has been shown to have a dramatic effect on early development of fishes. In fact at very early stages, anoxic conditions can pause development for 24 h with development resuming when normoxic conditions return (Padilla and Roth 2001); at later stages, hypoxia has been shown to cause developmental delay and in some cases to result in malformation of the body (reviewed by Wu 2009). Exposure to hypoxic conditions through development has been shown to change swimming performance in adult zebrafish (Widmer et al. 2006); periods of exposure in adults induce changes in the density of chemoreceptors of the gills and the ventilator response (Vulesevic et al. 2006). Along with development of the gills, the circulatory system is maturing in the transitional period, at about 3 weeks post-fertilization. After that time, the cardiovascular system becomes increasingly better at regulating oxygen consumption (Barrionuevo and Burggren 1999). Effects of hypoxia on morphology and movement of the pectoral fins have not been addressed in the transition during juvenile development, or in adult fish; however, probing respiratory development in conjunction with development of the fins offers opportunities to examine the coordination of development between body systems in response to environmental change.

## Conclusions

Developmental trajectories of organisms, from larval and juvenile forms to the full reproductive adult, provide a fascinating and useful framework for examining stability and change in biological structure, function, physiology, and ecology. A morphological system may be specialized to perform different roles for an organism through life history. The process of transition from one role to another may be accomplished in a number of ways and include periods in which a system is not particularly well adapted for any particular function. In some ways, transitions in body systems and their functions through development are analogous to changes among species in an evolutionary context. Evolutionarily, a movement system may be morphologically and/or physiologically adapted for alternative functions in different species. However, the process by which that functional diversity arose, which may involve a combination of changes in neural, muscular, skeletal, and other systems, is less obvious (e.g., Lauder 1981; Roth and Wake 1985). Understanding how organisms traverse periods of change between stable functions, whether through development or evolutionary history, provides insight into integrative organismal function and the pressures that shape morphological, physiological, and behavioral diversity.
